# Editors’ Book Table

**Published:** 1875-04

**Authors:** 


### Editors' Book Table.

[NOTE. — All works reviewed in the pages of the CHICAGO MEDICAL JOURNAL may be found in the extensive stock of W. B. KEEN, COOKE & CO., whose catalogue of Medical Books will be sent to any address upon request.]

PHYSICIAN'S CASE RECORD AND PRESCRIPTION BLANK BOOK, WITH VISITING LIST. Cincinnati Case Record Company. 1875.

Upon the first appearance of this Case Record as a candidate for popularity, we were not so much prepossessed in its favor as we have subsequently become by a practical acquaintance with its advantages. "The proof of the pudding is the eating thereof," and the same may be said of other things besides puddings. The use of these Case Records will enable the student, we mean the physician who studies his cases, to preserve a mass of clinical memoranda which would otherwise be lost. The Record has been found especially useful for the preservation of Notes of Hospital Practice, and for this purpose we have introduced it into St. Joseph's Hospital, where its advantages have been apparent to all. Some trouble is doubtless entailed by the use of any system of recording cases, and this will prove an insurmountable objection to all methods in the eyes of many. By these books, this trouble is reduced to a minimum, and, in comparison with the value of the object to be attained, becomes insignificant. H.

QUESTIONS IN ANATOMY: for the Use of Students. By *Corydon L. Ford, M.D.*, Professor of Anatomy in the University of Michigan, Ann Arbor, Mich. 1873.

This little book must prove very useful to students as a guide and indicator of what is necessary and useful to be learned in anatomy. The answers to the questions must be looked for elsewhere, in a text book, or in that best of all anatomical text books, the human body. The questions will serve very well to concentrate the attention of the student, and prevent waste of time upon incidental or irrelevant matter. An improvement suggests itself in the "make up" of the book, which we venture to hint to the author before the publication of a new edition, *i. e.*, the interpolation of blank pages upon which the student might write his answers as drawn from his own dissections, thus constituting it an invaluable aid to his memory, more permanent by far than his recollection of lessons in text books or oral instruction. H.

TRANSACTIONS OF THE PATHOLOGICAL SOCIETY OF PHILADELPHIA. Volume Fourth. Containing the Report of the Proceedings for the Years 1871–2–3. Edited by *James Tyson, M.D.*, Hospital Lecturer on Pathological Anatomy and Histology in the University of Pennsylvania, etc., Recorder of the Society. Philadelphia: Printed for the Society by J. B. Lippencott & Co. 1874.

A model volume, containing a List of Former Presidents, the Officers and Committees of the Society for the past year, a list of Members, comprising Non-Resident and Corresponding; and a List of Specimens presented to the Society, the Address of the Retiring President, and the Report of the Committee upon the Pathology of the various Morbid Specimens referred for Examination.

One feature in the list of members is somewhat novel, *i. e.*, the addendum, to certain names, "forfeited membership for non-payment of dues," which can scarcely be commended on the score of good taste, however efficient it may be as a protection to the finances of the Society.

The report upon each morbid specimen is brief, clear and explicit, based upon careful direct examinations, sustained, when necessary, by authority, but uncomplicated and unobscured by discussions and personal opinions.

The specimens represented number two hundred and forty-three—upon thirty-nine of which special reports have been made, of which nine are well illustrated by wood-cuts.

The whole constitutes the best specimen of a work of the kind we have ever seen, reflecting credit upon the Society at large, and especially upon the committees. The mechanical execution of this Report is very far above the average, and the paper, type and press work are creditable to the printers, Messrs. J. B. Lippencott & Co. H.

A Guide to the Practical Examination of Urine: for the Use of Physicians and Students. By *James Tyson, M.D.*, Hospital Lecturer on Pathological Anatomy in the University of Pennsylvania, etc., etc., with a plate and numerous illustrations. Philadelphia: Lindsay & Blakiston. 1875.

Dr. Tyson's name alone would be a guarantee of the excellence of a book. In this case, a careful examination fully justifies the assumption. The practical man will find in this little book all that is absolutely necessary for him to know, in order to utilize fully the data supplied by the urine, in the formation of accurate diagnoses, and will find it, moreover, presented in a condensed form, free from useless and irrelevant verbiage which sometimes encumbers more pretentious and elaborate treatises. A practitioner can hardly afford to dispense with this little manual, unless he has some equally good substitute, which would be difficult to find—a better we are sure he cannot find. H.

CLINICAL LECTURES ON DISEASES OF THE URINARY ORGANS. Delivered at University College Hospital by *Sir Henry Thompson*, Surgeon Extraordinary to His Majesty the King of the Belgians, Professor of Clinical Surgery, and Surgeon to the University College Hospital. Second American from the Third and Revised English Edition, with Illustrations. Philadelphia: Henry C. Lea. 1874.

Everything within the range of the pathology of the urinary organs, coming from the pen of Sir Henry Thompson, possesses interest and value. The volume before us comprises fourteen lectures essentially practical in their character, written clearly and concisely. The subjects are treated with the precision of a teacher, and the authority of a master, of one who has learned his art, and who never subordinates practical experience to theory. His instructions for passing the catheter or bougie are illustrative of this: "I pity the patient who has a solid instrument thrust into his body by a knowing man at anatomy," indicating thereby what is doubtless perfectly true, the necessity of paying more attention to the peculiarities of each individual urethra than to arbitrary anatomical rules.

For the treatment of stricture, the author gives the preference to internal incision, after describing fully and discussing fairly the processes of dilatation, gradual and forcible, and Symes', or the operation of external urethrotomy.

For the performance of the operation, too, he gives the preference to the instrument of Civiale, on account of its simplicity, as "far better than a machine which makes a certain incision mechanically, and is not controled or guided by the intelligence of the surgeon."

The chapters on prostatic disease are interesting. The distinction between the two forms, occurring at different periods of life, inflammatory enlargement, prostatitis, frequently observed in young subjects, and hypertrophy occurring in old age, is clearly drawn, each class of cases being well defined. The latter form of the disease the author declares to be by no means so frequent as has hitherto been asserted by writers, and generally believed by the profession at large. Out of two hundred persons dying beyond the age of fifty-five years in the Marylebone Infirmary, and dissected very carefully by himself, he found but three in one hundred to be the subjects of prostatic enlargement. He assumes, therefore, that one in ten would be a large estimate for the cases of disease of this organ in persons beyond the age of sixty years, which opinion will doubtless be sustained by the observation of the vast majority of his readers in this country at least.

In referring to the condition of the bladder as associated with stricture the author wishes "to render this sentence in the largest capitals," that "INVOLUNTARY MICTURITION INDICATES RETENTION AND NOT INCONTINENCE." The chapter upon Extravasation of Urine and Urinary Fistulæ, is written instructively and in a very hopeful spirit concerning the results of operative procedures in these distressing conditions.

With the subject of calculi and their removal, Sir Henry Thompson's name has been so thoroughly and universally identified, as to make whatever he may have to say upon it, of peculiar interest, and this assumption will only be justified by careful study of the chapter devoted to Lithotrity and Lithotomy. For the former operation, in the performance of which he has become so widely celebrated, his preference is indicated by the emphatic declaration, "if the stone is discovered when sufficiently small, *it can always be crushed with an almost* CERTAIN CHANCE *of success;*" "so that lithotomy for adults must at some day disappear, except for cases which have been neglected by patients themselves, or have been overlooked by the medical attendant."

Of the subject of Lithotomy there is a very interesting historical as well as surgical sketch which will well repay the reader.

One of the chief merits of this book is its brevity; what the author has to say is said well and concisely, the volume of less than two hundred pages containing more valuable matter than in many instances would be diluted with five or six hundred of words. The paper, type and mechanical work are better even than Mr. Lea's average standard, which is a high one. H.

Examinaton of the Urine. By *Geo. B. Fowler, M.D.*, Examiner in Physiology, College of Physicians and Surgeons, New York; Fellow of New York Academy of Medicine; Member of N. Y. County Medical Society, etc. New York: D. Appleton & Company. 1874.

"This book is intended simply as a Guide," says the author in his preface, and we are quite sure that the student who will make it his "guide" and follow its lead step by step through its exposition of the various conditions of urine, normal and morbid, and the means for their detection, will feel satisfied that his time has been profitably spent in studying Dr. Fowler's manual of eighty pages. H.

The Drift of Mental Philosophy. An Essay. By *D. A. Gorton, M.D.* Revised Edition. Philadelphia: J. B. Lippencott & Co. 1875.

This is a rehash of the "olla podrida" which was served up a year ago under the title "Principles of Mental Hygiene," and noticed somewhat at length in this Journal. Of the present (very unsuccessful) Essay we will borrow the criticism—truly Delphic—of the New York Medical Journal, "the work demands careful reading." H.

Inflammation of the Lungs: Tuberculosis and Consumption. Twelve Lectures. By *Dr. Ludwig Buhl*, Professor of Pathological Anatomy and General Pathology in the University of Munich, etc., etc., etc. Translated by permission from the Second German Edition, by Matthew D. Mann, M.D., and Samuel B. St. John, M.D. New York: G. P. Putnam's Sons. 1874.

In view of the great interest awakened in the subjects consumption and tuberculosis and the differential diagnosis, by the investigations of Rindfleisch, Niemeyer,

Schultze and others, these letters of Prof. Buhl must be read with peculiar pleasure, as the opinions of a pioneer in this field of research, whose opportunities for observation have been singularly favorable. The careful student will detect in the results of the author's investigations a striking similarity in the pathological processes occurring in the lungs to those observed by Johnson and others in the kidneys, and an illustration of the simplicity and comprehension of pathological—and, *a fortiori*, physiological—processes when isolated from incidental and considered only in relation to their essential factors. No one desirous to keep up with the advance of pulmonary pathology can afford to neglect this little book, and their thanks are especially due to the translators who have put the work so skillfully in the English garb for their use. H.

The Medical Uses of Alcohol; and Stimulants for Women. By *James Edmunds, M.D.*, Member of the Royal College of Physicians of London; Member of the Royal College of Surgeons of London; late Senior Physician to British Lying-in Hospital; Senior Physician to the London Temperance Hospital. New York: National Temperance Society and Publication House. 1874.

A temperance tract having for its essential characteristic, intemperance advocating temperance in intemperate language and appealing to reason by the shallowest sophistry—denouncing medical dogmata in opinions essentially dogmatic. This little book will, like millions of tons of its fellows, fail to accomplish its object for these very reasons. *Falsus in uno falsus in omnibus* will ever be the judgment of the human mind concerning books as well as men and principles.

### BOOKS RECEIVED.

The Pathological Significance of Nematode Entozoa. By *T. R. Lewis, M.B.*, Staff Surgeon to Her Majesty's British Forces, (on special duty), attached to the Sanitary Commissioner with the Government of India. Calcutta: Office of the Superintendent of Government Printing. 1874.

THE HISTOLOGY AND HISTO-CHEMISTRY OF MAN: a Treatise on the Elements of Composition and Structure of the Human Body, by *Heinrich Frey*, Professor of Medicine in Zurich. Translated from the Fourth German Edition, by Arthur E. J. Barker, Surgeon to the City of Dublin Hospital; Demonstrator of Anatomy, Royal College of Surgeons, Ireland; Visiting Surgeon, Convalescent Home, Stillorgan; and revised by the author. With six hundred and eight Engravings on Wood. New York: D. Appleton & Co. 1875.

FUNCTIONAL DERANGEMENTS OF THE LIVER, being the Croonian Lectures delivered at the Royal College of Physicians in March, 1874, by *Charles Murchison, M.D., LL.D., F.R.S.*, Fellow of the Royal College of Physicians; Physician and Lecturer on the Principles and Practice of Medicine, St. Thomas Hospital; Vice-President and Consulting Physician, London Fever Hospital; Formerly Physician and Lecturer on Medicine, Middlesex Hospital, and on the Medical Staff of H. M.'s Bengal Army. New York: William Wood & Co. 1875.

LECTURES ON THE DISEASES OF THE RESPIRATORY ORGANS, HEART AND KIDNEYS. By *Alfred Loomis*, Professor of Pathology and Practical Medicine in the Medical Department of the University of the City of New York; Consulting Physician to the Charity Hospital, etc., etc. New York: William Wood & Co. 1875.

A REPORT OF MICROSCOPICAL AND PHYSIOLOGICAL RESEARCHES into the Nature of the Agent or Agents Producing Cholera, (second series). By *T. R. Lewis, M.B.*, and *D. D. Cunningham, M.B.*, Surgeons, H. M. Indian Medical Service, (on special duty), attached to the Sanitary Commissioner with the Government of India. Calcutta: Office of the Superintendent of Government Printing. 1874.

CYCLOPÆDIA OF THE PRACTICE OF MEDICINE. Edited by *Dr. H. Von Ziemssen*, Professor of Clinical Medicine in Munich, Bavaria. Vol. II, Acute Infectious Diseases. By Prof. Thomas, of Leipzig; Dr. Curschmann, of Berlin; Dr. Leulzer, of Berlin; Prof. Hertz, of Amsterdam, and Prof. Von Ziemssen, of Munich. Translated by James C. White, M.D., and Edward Nigglesworth, Jr., M.D., of Boston; Edward W. Schauffler, M.D., of Kansas City; and A. Brayton Hall, M.D., J. Haven Emerson, M.D., George H. Fox, M.D., Edward Frankel, M.D., and John C. Jay, Jr., M.D., of New York. Albert H. Buck, M.D., of New York, Editor of American Edition. New York: Wm. Wood & Co. 1875.

### PAMPHLETS RECEIVED.

Scleritis Syphilitica: its Pathology, Course and Treatment. By *Fred. Sturges, M.D.*, one of the Surgeons to the Charity Hospital, Blackwell's Island, Lecturer on Venereal in the Medical Department of the University of New York, etc. New York: G. P. Putnam's Sons. 1875.

A Retrospect of the Struggles and Triumphs of Ovariotomy in Philadelphia, with some Additional Remarks on Allied Subjects: The Annual Address before the Philadelphia County Medical Society. By *Washington L. Atlee, M.D.*, Retiring President. Delivered Feb. 1, 1875. Published by order of the Society. Philadelphia. 1875.

Report of the Vaccine Department of the New York Dispensary for the year 1874. By *Frank P. Foster, M.D.*, Director of the Vaccine Department. New York. 1875.

Researches into the Action of Mercury, Podophyllin and Taraxacum on the Biliary Secretion, being the Report of the Edinburgh Committee of the British Association. By *John Hughes Bennett, M.D., F.R.S.E.*, Chairman and Reporter. Second Edition and Appendix. Chicago: C. H. Garrison, M.D. 1874.

Annual Announcement of the Medical College of the Pacific (Late Medical Department of the University of the Pacific), being the Medical Department of the University (City) College, San Francisco. Session of 1875.

Catalogue of the Trustees, Professors and Students of the Jefferson Medical College of Philadelphia. Session 1874–5. Also,

Graduates of the Jefferson Medical College of Philadelphia. March, 1875.

### JOURNALS RECEIVED.

The American Medical Weekly—Vol. ii, Nos. 8, 9, 10, 11, 12.
The Atlanta Medical and Surgical Journal—Vol. xii, Nos. 11, 12.
The American Practitioner—Vol. ii, No. 63.
The Boston Medical and Surgical Journal—Vol. xcii, Nos. 7, 8, 9, 10, 11.
The Canada Medical and Surgical Journal—Vol. iii, Nos. 8, 9.
The Clinic, Cincinnati—Vol. viii, Nos. 8, 9, 10, 11, 12.

The Cincinnati Lancet and Observer—Vol. xviii, No. 3.
The Canada Medical Record—Vol. iii, No. 7.
The Detroit Review of Medicine and Pharmacy—Vol. x, No. 3.
The Eclectic Medical Journal—Vol. xxxv, No. 3.
The Indiana Journal of Medicine—Vol. i, No. 11.
The Journal of Materia Medica—Vol. xiv, No. 2.
The Kansas City Medical Journal—Vol. v, No. 1.
The London Lancet—February, 1875.
The Monthly Abstract of Medical Sciences—Vol. ii, Nos. 2, 3.
The Medical and Surgical Reporter, Philadelphia—Vol. xxxii, Nos. 8, 9, 10, 11, 12.
The Medical Times, Philadelphia—Vol. v, Nos. 173, 174, 175, 176, 177.
The Medical Herald, Leavenworth—Vol. viii, Nos. 8, 9.
The Medical Record, New York—Vol. x, Nos. 8, 9, 10, 11, 12.
The Medical Examiner, Chicago—Vol. xvi, No. 5.
The Medical News and Library—Vol. xxxiii, No. 387.
The Missouri Clinical Record—Vol. i, No. 12.
The Medical Eclectic—Vol. ii, No. 2.
The Medical Press and Circular, London—No. 1880.
The Nashville Journal of Medicine and Surgery—Vol. xv, No. 3.
The New York Medical Journal—Vol. xxi, No. 3.
The New Orleans Medical and Surgical Journal—Vol. ii, No. 5.
The Ohio Medical and Surgical Reporter—Vol. ix, No. 2.
The Pacific Medical and Surgical Journal—Vol. xvi, Nos. 9, 10.
Le Progres Medical, 3c Annee—Nos. 1, 2, 3, 4, 5.
The Physician and Pharmacist—Vol. viii, No. 1.
The Practitioner, London—No. lxxx.
The Pharmacist—Vol. viii, No. 3.
The Psychological and Medico-Legal Journal—Vol. ii, No. 3.
The Peninsular Journal of Medicine—Vol. xi, No. 3.
The Richmond and Louisville Medical and Surgical Journal—Vol. xix, No. 23.
El Repertorio Jalisciense, Guadalajara—Tomo i, No. 7, 8.
The Southern Medical Record—Vol. v, No. 1.
The St. Louis Medical and Surgical Journal—Vol. xii, Nos. 2, 3.
The Technologist—Vol. vi, No. 2.
The United States Medical Investigator—Vol., Nos. 1, 4.
The Virginia Medical Monthly—Vol. i, No. 12.

